# Oral Microbiome Dysbiosis Is Associated With Symptoms Severity and Local Immune/Inflammatory Response in COVID-19 Patients: A Cross-Sectional Study

**DOI:** 10.3389/fmicb.2021.687513

**Published:** 2021-06-23

**Authors:** Irene Soffritti, Maria D’Accolti, Chiara Fabbri, Angela Passaro, Roberto Manfredini, Giovanni Zuliani, Marco Libanore, Maurizio Franchi, Carlo Contini, Elisabetta Caselli

**Affiliations:** ^1^Section of Microbiology, CIAS Research Center and LTTA, Department of Chemical and Pharmaceutical Sciences, University of Ferrara, Ferrara, Italy; ^2^Section of Dentistry, Department of Biomedical and Specialty Surgical Sciences, University of Ferrara, Ferrara, Italy; ^3^Unit of Internal Medicine, Department of Translational Medicine, University of Ferrara, Ferrara, Italy; ^4^Medical Clinic Unit, Department of Medical Sciences, University of Ferrara, Ferrara, Italy; ^5^Unit of Infectious Diseases, University Hospital of Ferrara, Ferrara, Italy; ^6^Section of Infectious Diseases and Dermatology, Department of Medical Sciences, University of Ferrara, Ferrara, Italy

**Keywords:** oral microbiome, COVID-19, symptom severity, inflammatory cytokines, secretory IgA

## Abstract

The human oral microbiome (HOM) is the second largest microbial community after the gut and can impact the onset and progression of several localized and systemic diseases, including those of viral origin, especially for viruses entering the body via the oropharynx. However, this important aspect has not been clarified for the new pandemic human coronavirus SARS-CoV-2, causing COVID-19 disease, despite it being one of the many respiratory viruses having the oropharynx as the primary site of replication. In particular, no data are available about the non-bacterial components of the HOM (fungi, viruses), which instead has been shown to be crucial for other diseases. Consistent with this, this study aimed to define the HOM in COVID-19 patients, to evidence any association between its profile and the clinical disease. Seventy-five oral rinse samples were analyzed by Whole Genome Sequencing (WGS) to simultaneously identify oral bacteria, fungi, and viruses. To correlate the HOM profile with local virus replication, the SARS-CoV-2 amount in the oral cavity was quantified by digital droplet PCR. Moreover, local inflammation and secretory immune response were also assessed, respectively by measuring the local release of pro-inflammatory cytokines (L-6, IL-17, TNFα, and GM-CSF) and the production of secretory immunoglobulins A (sIgA). The results showed the presence of oral dysbiosis in COVID-19 patients compared to matched controls, with significantly decreased alpha-diversity value and lower species richness in COVID-19 subjects. Notably, oral dysbiosis correlated with symptom severity (*p* = 0.006), and increased local inflammation (*p* < 0.01). In parallel, a decreased mucosal sIgA response was observed in more severely symptomatic patients (*p* = 0.02), suggesting that local immune response is important in the early control of virus infection and that its correct development is influenced by the HOM profile. In conclusion, the data presented here suggest that the HOM profile may be important in defining the individual susceptibility to SARS-CoV-2 infection, facilitating inflammation and virus replication, or rather, inducing a protective IgA response. Although it is not possible to determine whether the alteration in the microbial community is the cause or effect of the SARS-CoV-2 replication, these parameters may be considered as markers for personalized therapy and vaccine development.

## Introduction

The human oral microbiome (HOM) is the second largest and complex microbial community after that of the gut in the human body (Wade, 2013; Caselli et al., 2020a). HOM dysbiosis is often associated with periodontal inflammation and has been reportedly associated with several local and systemic disease conditions (Baghbani et al., 2020; Caselli et al., 2020a), including those sustained by viral infections (Cagna et al., 2019; Baghbani et al., 2020). Indeed, the role of HOM in the establishment of the infection of many viruses entering the body via the oropharynx has been reportedly recognized (Baghbani et al., 2020). The microbial component of a eubiotic HOM can inhibit pathogen colonization by competitive exclusion and/or by empowering the immune response (Wilks et al., 2013). There is evidence that crucial mutual interactions occur between viruses and the microbiome (Wilks and Golovkina, 2012) and that the microbiome can regulate and is in turn regulated by viruses via different mechanisms (Li et al., 2019). Respiratory viruses spread by aerosol transmission encounter oral and upper respiratory microbiota and are modulated in their ability to establish infection and able to induce changes in the resident microbiota (Li et al., 2019). The microbiota can produce antiviral compounds (defensins) against several viruses, including respiratory or oral viruses such as adenoviruses, herpesviruses, papillomaviruses, orthomyxoviruses, and coronaviruses (Pfeiffer and Sonnenburg, 2011). On the other hand, viruses can alter the microbiota, favoring dysbiosis and disease progression (Lynch, 2014).

The new pandemic human coronavirus SARS-CoV-2, causing COVID-19 disease, is a respiratory virus that uses the oropharynx as the primary site of replication, but the potential impact of HOM in the development of infection is still not elucidated. In particular, no data are available about the non-bacterial components of the HOM (fungi, viruses), which have been shown crucial for other diseases. Concerning the current pandemics by SARS-CoV-2, the presence of gingival inflammation/periodontitis has been associated with a 3.5-fold increased risk of admission to intensive care units (ICU), a 4.5-fold greater risk of assisted ventilation, and a consistent impressive 8.81-fold higher risk of death in COVID-19 patients, independently from other concomitant risk factors (Marouf et al., 2021).

The novel human Severe Acute Respiratory Syndrome Coronavirus type (SARS-CoV-2) is a single strand RNA virus belonging to the *Coronaviridae* family, β-coronavirus genus (Contini et al., 2020), which has spread worldwide. The associated disease, Corona Virus Disease 2019 (COVID-19), is currently reported by the World Health Organization (WHO) to have caused about 120 million cases with >2.6 million deaths (World Health Organization [WHO], 2021). In Italy, to date over 3.2 million cases have been reported, with other 102,000 deaths. The disease is characterized by the involvement of the lower respiratory tract, often accompanied by elevated blood levels of inflammatory cytokines/chemokines, the so-called “cytokine storm” (de la Rica et al., 2020; Jose and Manuel, 2020), by ageusia and/or hyposmia (Contini et al., 2020; Li et al., 2020; Prasad et al., 2020), and neurological and enteric symptoms in severely symptomatic patients (Contini et al., 2020; Gupta et al., 2020).

An extraordinarily high number of studies were published the last year, yet the mechanisms underlying virus proliferation in the primary site of infection and understanding of how the virus can become more invasive at the site of entry is still unclear, even though this could shed important light on the very first phases of the infection. It is recognized that SARS-CoV-2 enters the body mainly via the oropharynx, where it finds epithelial cells expressing the ACE2 and TMPRSS2 virus receptors (Herrera et al., 2020), and the virus has been detected in saliva (Henrique Braz-Silva et al., 2020; To et al., 2020). Thus, the resident oral microbiome may influence the ability of SARS-CoV-2 to take root and establish the infection. Similar to what is reported for other viruses affecting the oral and respiratory tract, the virus-host interplay in this site may define the vulnerability of the infected subject and the subsequent development of the disease or rather the early control of virus infection and prevention of severe disease. Like other microbial communities in the body, the oral microbiome can represent a protective barrier against exogenous pathogens (Zaura et al., 2009; Wade, 2013; He et al., 2015; Deo and Deshmukh, 2019) and it contributes to the lung microbiome, thus potentially affecting also the microbial environment in the lungs (Bassis et al., 2015). The oral microbiome can contribute to regulating mucosal immunity and inflammation, which might affect pathogenic potential directly or indirectly (Belkaid and Hand, 2014; Lamont et al., 2018).

Although there is potential interest in understanding these networks in SARS-CoV-2 infection, no information is yet available about the microbiome profile in COVID-19 patients, except for a two as yet unpublished reports describing the bacterial component of the oral microbiome by NGS (Iebba et al., 2020; Ward et al., 2021). However, several reports have evidenced that the non-bacterial components of the microbiome can be very important in defining individual susceptibility to diseases besides bacteria (the mycome and virome), thus the use of Whole Genome Sequencing (WGS) technology may be more useful in elucidating the microbial environment potentially impacting on SARS-CoV-2 infecting ability.

The present work aimed to characterize, for the first time, the oral microbiome of COVID-19 patients by WGS, comparing its profile to controls, and simultaneously evaluating the presence of inflammatory cytokines and local IgA immune response, to better understand the features of the oral environment that could potentially support SARS-CoV-2 infection and related disease, and to identify eventual markers for the risk of developing a severe infection.

## Materials and Methods

### Ethics Statement

Recruitment of study participants was performed according to the protocol approved by the Ethics Committee Area Vasta Emilia Centro della Regione Emilia-Romagna (CE-AVEC): approval document no. 408/2020/Oss/UniFe, approved on April 21, 2020.

### Design of the Study

A cross-sectional observational study was performed to characterize the oral microbiome and local response in COVID-19 patients compared to non-COVID-19 subjects. All participants were recruited from the University Hospital of Ferrara, in the COVID-19 and the non-COVID Infectious ward, respectively. Study participants were recruited in the period April to July 2020. Each study participant was recruited after signing informed consent. Clinical and epidemiological data were collected from the clinicians of the enrolled ward. The study was registered and published prospectively in the ISRCTN International Registry (study n° ISRCTN87832712; doi: 10.1186/ISRCTN87832712).

### Study Participants

All study participants were recruited among the hospitalized patients of the University Hospital of Ferrara. Inclusion criteria were: age >18 years, written consent to participate in the study, and molecular diagnosis of SARS-CoV-2 infection (for COVID-19 group only). Exclusion criteria included: pregnancy, breastfeeding, uncooperative patient (inability to perform oral rinse to collect samples), lack of written agreement. COVID-19 patients were stratified into four categories based on symptoms: asymptomatic (1, no symptoms), paucisymptomatic (2, aspecific flu-like symptoms), symptomatic (3, including specific respiratory symptoms), severely symptomatic (4, needing ventilation). The control group consisted of SARS-CoV-2-negative subjects affected by non-respiratory diseases. The number of study participants was decided based on the subjects hosted at the University Hospital of Ferrara in the study period.

### Clinical Specimens

Oral rinse samples were collected in 5 mL of sterile phosphate-buffered saline (PBS), as previously described (Caselli et al., 2020a). The specimens were immediately inactivated with 0.1% SDS, refrigerated (2–8°C), and processed within 4 h. Briefly, all samples were vortexed and centrifuged at 15,000 × *g* for 10 min at 4°C to divide the corpuscular part from the supernatant, which were immediately frozen in liquid nitrogen and kept at −80°C until use.

### Nucleic Acid Extraction From Clinical Specimens

Total nucleic acids (DNA and RNA) were extracted from the pellets by using the Maxwell CSC platform equipped with the HT Viral TNA Kit (Promega, Milan, Italy), following the manufacturer’s instructions (Comar et al., 2019). Extracted total nucleic acids (TNAs) were checked and quantified by nanodrop spectrophotometric (Thermo Fisher Scientific, Milan, Italy) reading at 260/280 nm. The amplificability of extracted DNA was checked by PCR amplification of human, bacterial, and fungal genes. Namely, human β-actin, bacterial 16S rRNA gene (pan bacterial PCR, *panB*), and mycetes ITS gene (pan fungal PCR, *panF*) were respectively, analyzed, as previously described (Borghi et al., 2016; Caselli et al., 2016, 2018).

### Library Preparation and Sequencing

Extracted TNA (100 ng) were retrotranscribed and analyzed by WGS by the NGS Service of the University of Ferrara (Department of Morphology, Surgery and Experimental Medicine, University of Ferrara), who carried out library preparation, sequencing, and taxonomic analysis. Briefly, WGS libraries were prepared using NEBNext^®^ Fast DNA Fragmentation and Library Prep Kit for Ion Torrent TM (Thermo Fisher Scientific, Milan, Italy), following the manufacturer’s protocol. Samples were then sequenced by using the Ion Gene Studio S5 System (Thermo Fisher Scientific, Milan, Italy). Low-quality sequence data removal was performed directly on the Ion S5 GeneStudio sequencer, as part of in-built processing. Briefly, the Torrent Suite software (Thermo Fisher Scientific, Milan, Italy), installed in the sequencer, automatically clips adapter sequences and trims low-quality bases from the 3′ end of each read. Reads with quality less than Q20 were also discarded. Additionally, PRINSEQ open source application (Schmieder and Edwards, 2011) was used to remove reads with lengths of less than 100 nucleotides. The taxonomic assignment has been performed using Kraken2 (Pubmed ID: 24580807) and a database consisting of archaea, bacteria, fungi, protozoa, and viruses. Raw sequencing data and bioinformatics analyses have been deposited in the European Nucleotide Archive (ENA) website (accession number PRJEB42999).

### SARS-CoV-2 Detection and Quantification

Extracted TNA (100 ng) was used for SARS-CoV-2 detection and quantification by droplet digital PCR (ddPCR), by using the SARS-CoV-2 ddPCR Kit (Bio-Rad Laboratories, Milan, Italy). Briefly, three targets are analyzed in each sample by FAM and HEX labeled probes, targeting SARS-CoV-2 N1 and N2 genes, and human RPP30 gene, this last was used as a control and to normalize the virus counts. The assay sensitivity was between 0.260 copies/μl to 0.351 copies/μl, respectively, for the genetic markers N1 and N2.

### IgA Analysis

The presence of anti-SARS-CoV-2 secretory IgA (sIgA) in the oral samples was evaluated by a CE-IVD ELISA assay designed to detect IgA directed against the virus S1 protein (Euroimmun, Lubeck, Germany). The test was previously reported to have high specificity/sensitivity for IgA detection in serum/plasma samples (>95%) and ocular fluids (Caselli et al., 2020b). For oral rinse analysis, the samples were diluted 1:5 in saline, allowing optimal detection of IgA and differentiation between positive samples and controls, as detected in preliminary assays. Each sample was assessed in triplicate. Sample positivity was expressed following the manufacturer’s instruction, as the ratio (R) between the absorbance (OD_450 *nm*_) value detected in samples and that detected in the calibrator sample provided by the manufacturer. Samples were considered negative if *R* values were < 0.8, weakly positive with *R* values comprised between 0.8 and 1.1, and strongly positive with R ≥ 1.1.

### Cytokines Analysis

Oral specimens were analyzed for the presence of pro-inflammatory cytokines, by using ELISA assays specifically detecting and quantitating the following cytokines: IL-6, IL-17, TNFα, and GM-CSF (Thermo Fisher Scientific, Life-Technologies, Milan, Italy).

### Statistical Analyses

Statistical analyses were performed with Agilent GeneSpring GX v11.5 software (Agilent Technologies, Santa Clara, CA, United States) and R (R 2019, R Core Team, available as free software at https://www.r-project.org/). Microbiome data were expressed as the relative abundance of each taxonomic unit at the genus or species level. The null hypothesis was tested by the Kruskal–Wallis test. Pairwise *post hoc* analysis was performed by the non-parametric Dunn test which includes correction for multiple comparisons. A Chi-square test was used to assess gender distribution significance. Alpha-diversity obtained by measuring the Shannon H’ diversity index was used to describe the microbiome diversity between clinical samples. ELISA results were analyzed by Student’s *t*-test. Linear regression and correlation analyses (Spearman *r* correlation coefficient) were conducted to evaluate the correlation between patients’ clinical parameters (a non-continuous discrete variable), and continuous variables including microbiome profile, immune and inflammation responses. A *p*-value ≤ 0.05 was considered significant.

## Results

### Patients’ Characteristics

Seventy-five eligible subjects, including 39 COVID-19 patients and 36 controls, were enrolled in the study. COVID-19 patients included 20 males (51.3%) and 19 females (48.7%), with a mean age of 71.1 ± 18.4 years (range 25–99). Oral rinses were collected from COVID-19 patients at 0–43 days since the first SARS-CoV-2-positive nasopharyngeal swab. At the time of sample collection, 11/39 (28.2%) COVID-19 patients were asymptomatic, 7/39 (17.9%) presented mild symptoms, 21/39 (55.3%) were symptomatic, with 2 of them (2/39, 5.1%) showing severe respiratory symptoms requiring ventilation. All recruited COVID-19 patients received hydroxychloroquine and azithromycin on hospitalization (Gautret et al., 2020). The control group consisted of SARS-CoV-2-negative subjects admitted for non-respiratory diseases at the non-COVID Infectious Disease ward, and included 22 males and 14 females (respectively, 61% and 39% of the group), with a mean age of 66.5 ± 18.8 years (range 20–94). The characteristics of study participants are reported in Table 1. No statistical differences were evidenced between COVID-19 and control group with regard to age (Kruskal–Wallis test; *p* = 0.27, n.s.) and gender (Chi-square test; χ^2^ = 0.734, *p* = 0.39, n.s.). Similarly, no statistically significant differences were evidenced between COVID-19 disease sub-groups (asymptomatic, pauci-symptomatic, and symptomatic) regarding age (Kruskal–Wallis test; *p* = 0.21, n.s.) or gender distribution (Chi-square test; χ^2^ = 0.256, *p* = 0.88, n.s.).

**TABLE 1:** Characteristics of COVID-19 and control study participants.

Subject n°	Control group		COVID-19 group				Age/gender distribution
	Gender	Age	Gender	Age	Days after NPS	COVID-19 symptoms (*)	
1	F	74	M	76	13	3	***Age:***
2	M	72	F	72	4	3	**CTR:** 66.5 ± 18.8 years
3	M	73	F	56	0	1	**COVID-19:** 71.1 ± 18.4 years
4	F	86	M	49	3	1	**CTR vs. COVID-19:** *p* = 0.27, n.s.
5	F	38	F	49	6	2	
6	F	66	M	99	6	2	
7	F	67	F	80	18	4	
8	F	40	F	73	16	3	
9	M	53	F	68	2	1	
10	M	42	F	33	18	2	
11	M	75	F	51	2	2	
12	F	86	M	76	4	3	
13	M	60	M	82	8	3	
14	M	59	F	87	29	1	
15	M	83	M	47	6	1	
16	M	86	F	91	18	3	
17	F	86	M	89	5	3	

18	M	71	F	94	16	3	***Gender:***
19	M	88	M	94	20	2	**CTR:** 22/36 males (61%)
20	M	84	M	80	15	3	**COVID-19:** 20/39 males (51.3%)
21	F	88	M	85	18	3	**CTR vs. COVID-19:** *p* = 0.39, n.s.
22	F	86	F	83	7	3	
23	M	20	M	25	10	3	
24	F	94	F	78	18	4	
25	F	46	F	83	49	3	
26	F	76	F	45	17	3	
27	M	50	M	82	1	1	
29	M	51	F	82	2	1	
30	M	53	M	59	0	2	
31	M	45	M	45	43	1	
32	M	70	M	57	5	3	
33	F	85	F	86	4	3	
34	M	49	F	48	3	2	
35	M	67	M	90	11	3	
36	M	49	F	70	0	1	
37	M	76	M	81	51	1	
38	–	–	M	87	23	3	
39	–	–	M	78	11	1	
	–	–	M	63	18	3	

(*) Symptom score was: 1, asymptomatic; 2, paucisymptomatic; 3, symptomatic; 4, severely symptomatic.

*NPS, nasopharyngeal swab.*

*Age and gender distribution significance were assessed, respectively, by Kruskal–Wallis and Chi-square tests.*

### SARS-CoV-2 Load in COVID-19 Patients

**FIGURE 1:**
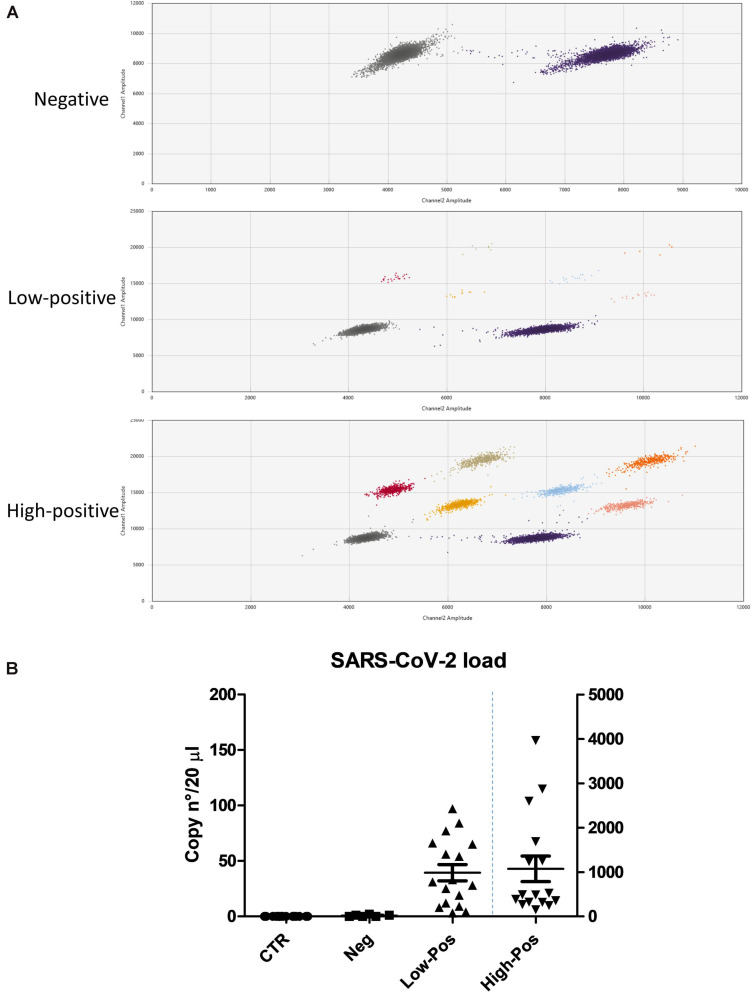
SARS-CoV-2 load in control and COVID-19 subjects, as measured by ddPCR. **(A)** Graphical representation of the values detected by the use of three different molecular probes: negative samples, only the clouds corresponding to the housekeeping control genes are detectable (gray and purple clouds); low- and high-positive samples, the clouds corresponding to the virus genes are detectable and counted (positives to individual FAM probes: gray, red, and yellow; positives to individual HEX probes: purple, blue, and pink; double positives to FAM/HEX probes: beige and orange). **(B)** Virus load, expressed as genome copy number per analyzed sample (20 μl of extracted nucleic acid); left *y* axis refers to control, negative and low-positive values, whereas right *y* axis refers to high-positive COVID-19 subjects. Mean value ± SEM is also reported.

Although all the enrolled COVID-19 patients were confirmed to be SARS-CoV-2 positive at hospital admission by the routine molecular test performed on nasopharyngeal swab by the Hospital microbiology laboratory, we wanted to assess the presence of SARS-CoV-2 in the oral cavity of all the enrolled subjects at the time of oral rinse withdrawal. The oral rinse samples were analyzed by digital droplet PCR (ddPCR), able to detect and quantify the virus genomes, contrarily to the routinely used diagnostic assays (Falzone et al., 2020; Suo et al., 2020). While the results confirmed the absence of positivity in the control group, in the COVID-19 group both positive and negative oral rinse specimens were observed, as summarized in Figure 1. Quantitative analysis showed that 16/39 subjects harbored a high load of SARS-CoV-2 (from 101 to 3,963 genome copies in 20 μl of the amplified sample), 17/39 had lower but detectable amounts of virus (from 3 to 100 genome copies in 20 μl), whereas 6/39 patients did not display any detectable virus copy in the oral cavity at the time of oral withdrawal (<3 copies in 20 μl). It is noteworthy that the virus load detected in the oral cavity correlated with symptom severity (Spearman *r* = 0.774; 95% CI 0.608–0.875) (*p* < 0.0001), defining specific subpopulations of COVID-19 patients.

### Oral Microbiome in COVID-19 Patients

**FIGURE 2:**
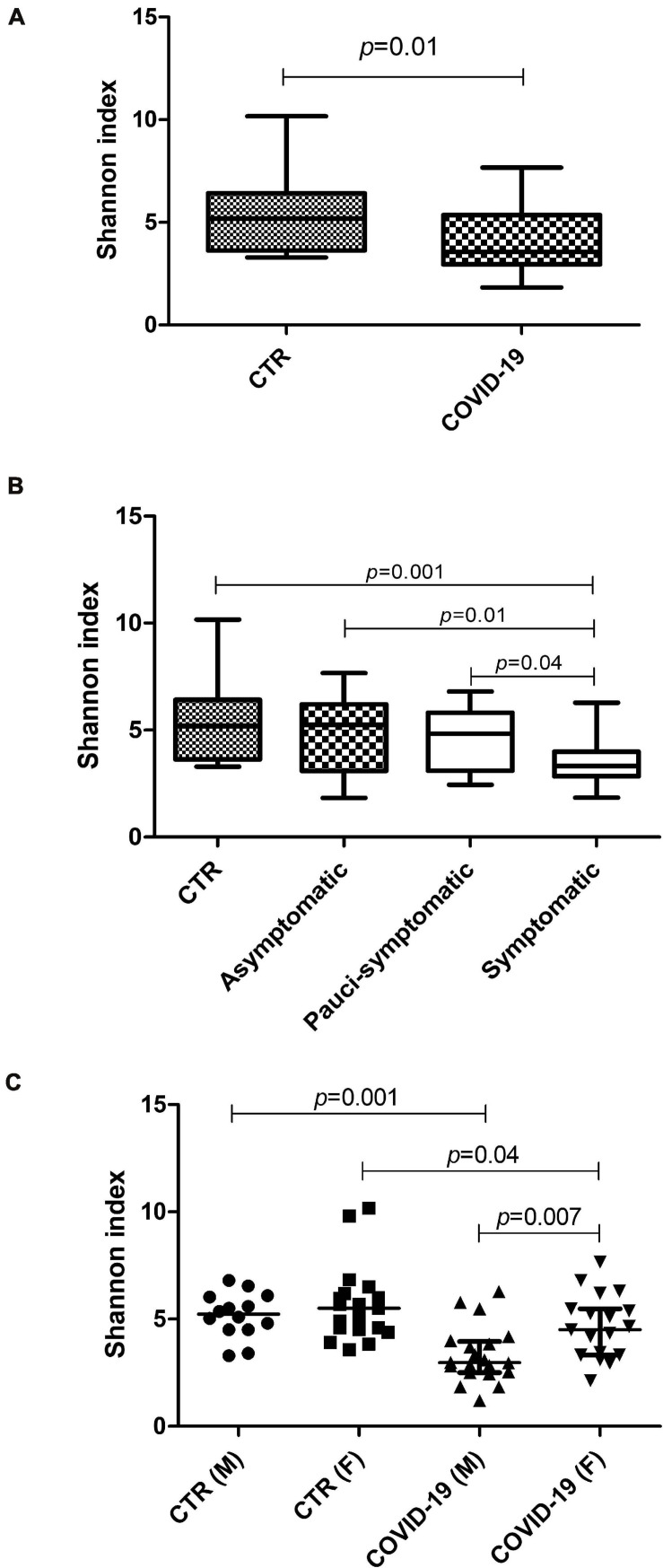
Alpha-diversity values in the oral microbiomes of control and COVID-19 subjects. **(A)** Comparison between control and COVID-19 patients, expressed as median and range values. **(B)** Comparison between controls and COVID-19 asymptomatic, pauci-symptomatic, and symptomatic subjects; median and range values are shown. **(C)** Comparison between genders (M, male; F, female) in the control and COVID-19 groups; median values with interquartile range are shown for each group.

Whole Genome Sequencing analysis of the oral microbiome evidenced significant differences in the profiles of the COVID-19 compared to controls. Alpha-diversity values were lower in COVID-19 patients vs. controls (*p* = 0.01) (Figure 2A). Interestingly, the comparison between severely symptomatic COVID-19 subgroups and controls revealed the most significant differences (Figure 2B), with an inverse correlation between alpha-diversity value and symptoms (Spearman *r* = −0.431, 95% CI −0.666/−0.120, *p* = 0.006). The decrease of alpha diversity was higher in male compared to female patients (Figure 2C), which paralleled symptoms severity.

**FIGURE 3:**
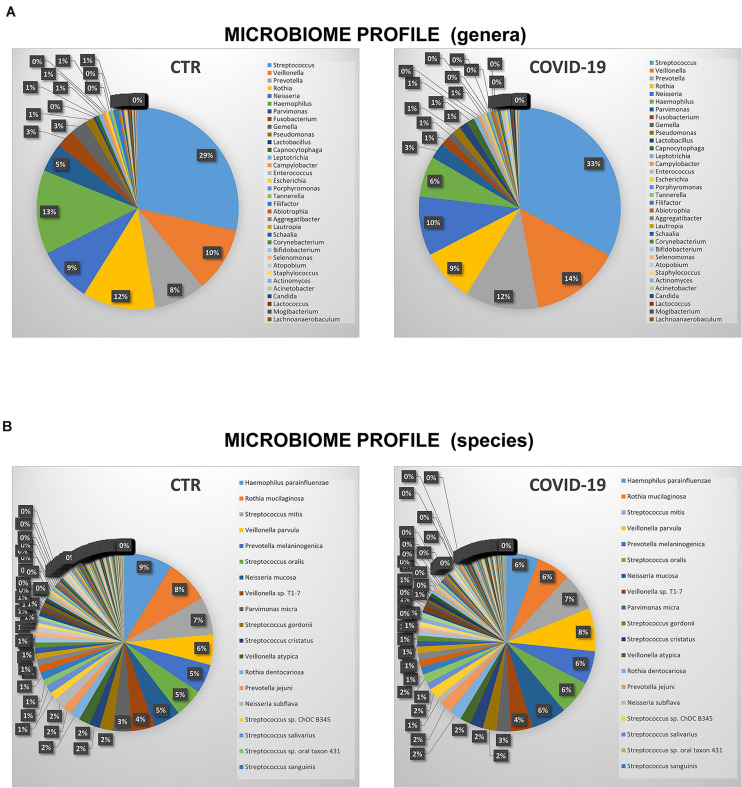
Relative abundance and distribution of microorganisms in the oral cavity of control (CTR) and COVID-19 subjects. **(A)** Percentage distribution of most detected microbial genera. **(B)** Percentage distribution of most detected microbial species.

**FIGURE 4:**
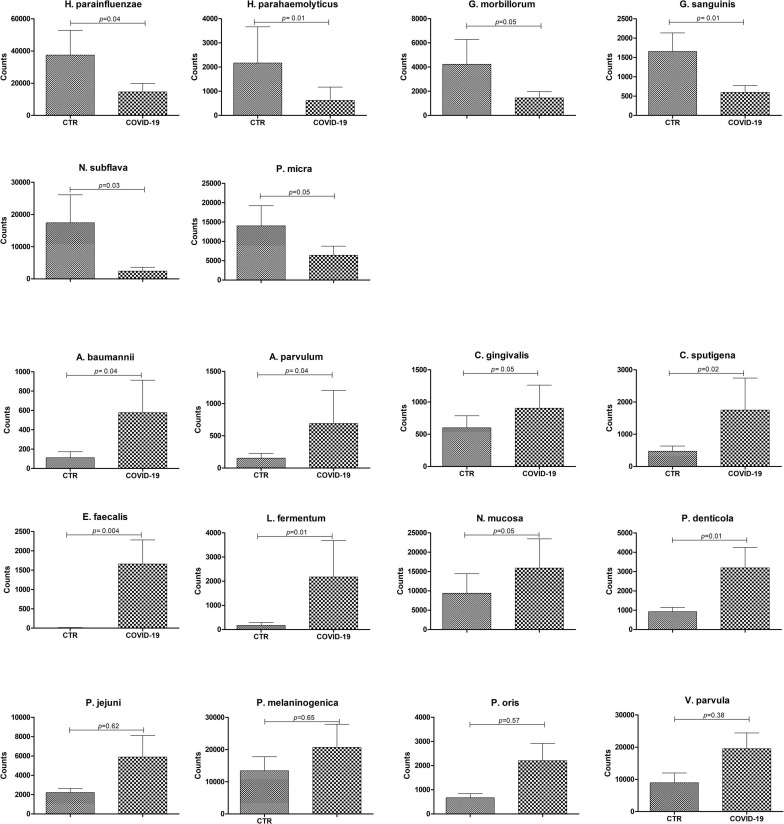
Significantly altered taxa in control (CTR) and COVID-19 subjects. The results are expressed as normalized counts ± SEM values. Significance *p*-values for each comparison are also displayed.

The microbiome profile appeared profoundly altered in COVID-19 patients compared to controls (Figure 3). In particular, the relative abundance of the bacterial genera *Streptococcus*, *Veillonella*, *Prevotella*, *Lactobacillus*, *Capnocytophaga, Porphyromonas, Abiotrophia, Aggregatibacter, Atopobium* was increased in COVID-19 compared to controls, whereas *Rothia*, *Haemophilus*, *Parvimonas*, *Fusobacterium*, and *Gemella* spp. were decreased (Figure 3A). Notably, *Enterococcus* and *Enterobacter* genera were exclusively present in COVID-19 patients, and not detectable in control subjects. At the species level (Figure 3B), COVID-19 patients had decreased amounts of *Haemophilus parainfluenzae* and *parahaemolyticus*, *Gemella morbillorum* and *sanguinis*, *Parvimonas micra*, and *Neisseria subflava*, whereas *Neisseria mucosa*, *Veillonella parvula*, *Lactobacillus fermentum*, *Enterococcus faecalis*, *Atopobium parvulum*, *Acinetobacter baumannii* were increased. Notably, many species of periodontopathogenic bacteria (*Prevotella melaninogenica*, *jejuni*, *denticola*, and *oris*; *Eikenella corrodens*; *Capnocytophaga sputigena* and *gingivalis*; and *Aggregatibacter aphrophilus*) were significantly increased in COVID-19 compared to control subjects. Figure 4 summarizes the taxa significantly altered in COVID-19 patients compared to controls, with significance values.

**FIGURE 5:**
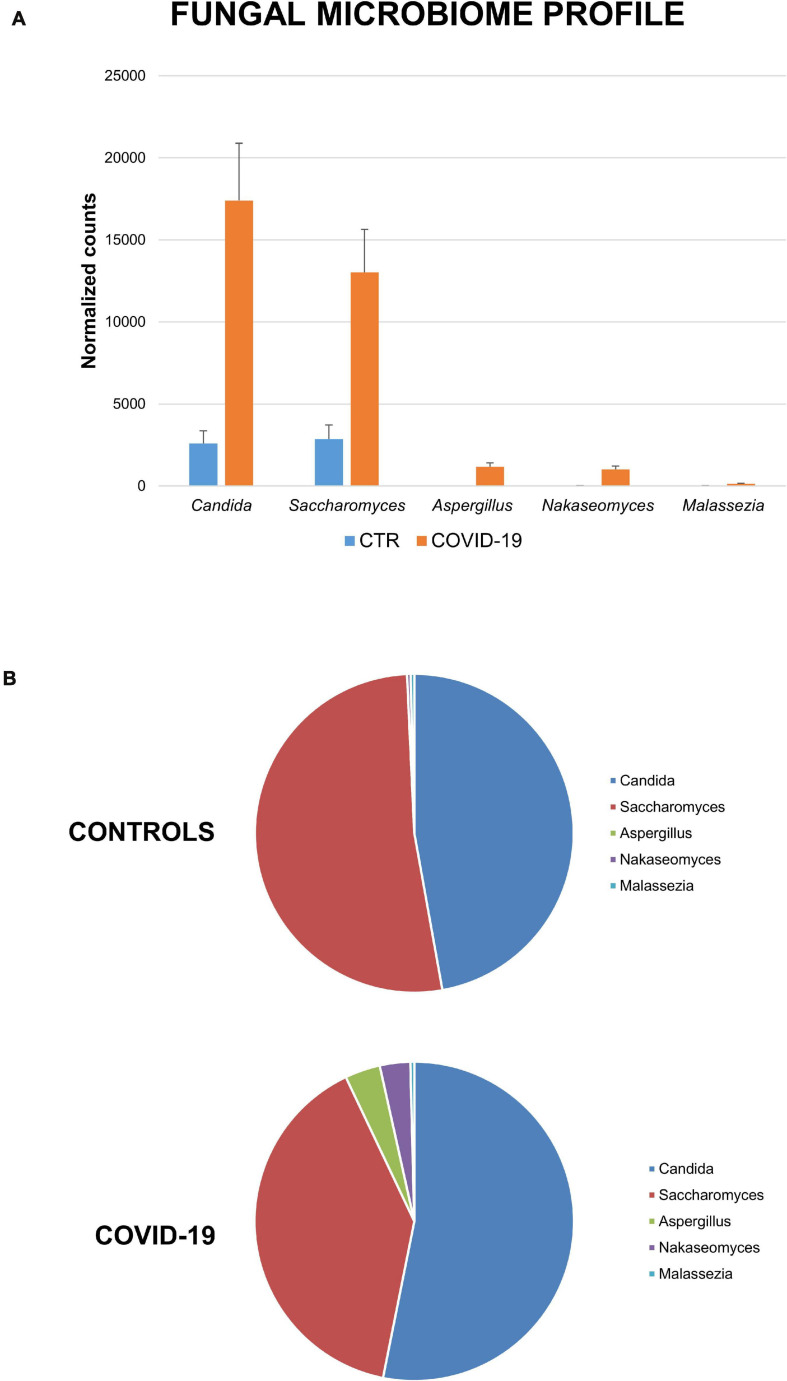
Mycome profile in the oral cavity of control and COVID-19 subjects. **(A)** Abundance of fungi expressed as total normalized counts for each individually detected mycetes. **(B)** Percentage distribution of the fungal genera in controls and COVID-19 patients.

It is of note that high differences were observed relative to the fungal component of the oral microbiome (Figure 5). Contrary to the decreased richness of the bacterial component, the fungal fraction of the oral microbiome was increased in COVID-19 patients compared to controls, both as total normalized counts and as species richness. In detail, while the oral mycobiome of controls was essentially constituted by *Candida* and *Saccharomyces* spp. (47% and 52% of relative abundance, respectively), in COVID-19 patients *Aspergillus*, *Nakaseomyces*, and *Malassezia* spp. were detectable at a fair by *Candida* and *Saccharomyces* spp. (47 and 52% of relative abundance, respectively), in COVID-19 patients *Aspergillus*, *Nakaseomyces*, and *Malassezia* spp. were detectable at a fair level, with respective relative abundance values of 4%, 3%, and <1%. The species *Candida albicans*, *Saccharomyces cerevisiae*, *Aspergillus fumigatus*, and *Malassezia restricta* were identified.

**FIGURE 6:**
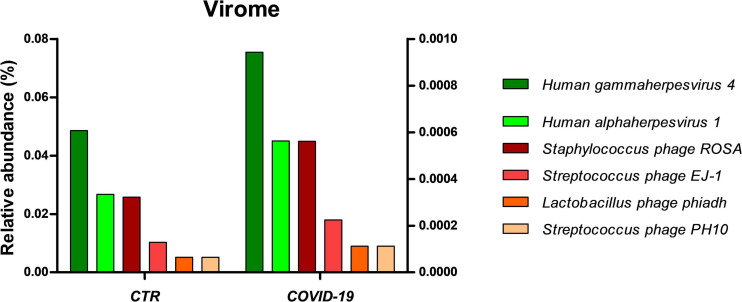
Virome profile in the oral cavity of control (CTR) and COVID-19 subjects. The results are expressed as relative abundance (%). Left *y* axis refers to Human gammaherpesvirus 4 and Human alphaherpesvirus 1 (green bars), whereas right *y* axis refers to the amount of the four detected bacteriophage (orange-red bars).

Interestingly, the oral virome also appeared more abundant in COVID-19 patients compared to controls (Figure 6). While viruses represented 0.07% of the microbial community in controls, their relative abundance increased to 1.12% in COVID-19 patients. *Lymphocryptovirus* and *Simplexvirus* genera of the Herpesviridae family were detected both in COVID-19 and control subjects. However, Epstein Barr virus (EBV) resulted reactivated in 11/39 COVID-19 patients and in only 2/36 controls. Moreover, Herpes simplex virus type 1 (HSV-1) and four bacteriophages targeted, respectively, toward *Staphylococcus* (Staphylococcus phage ROSA), *Streptococcus* (Streptococcus phage EJ-1 and phage PH10), and *Lactobacillus* (Lactobacillus phage phiadh), were also increased in COVID-19 patients compared to controls.

### Oral IgA Response in COVID-19 Patients

**FIGURE 7:**
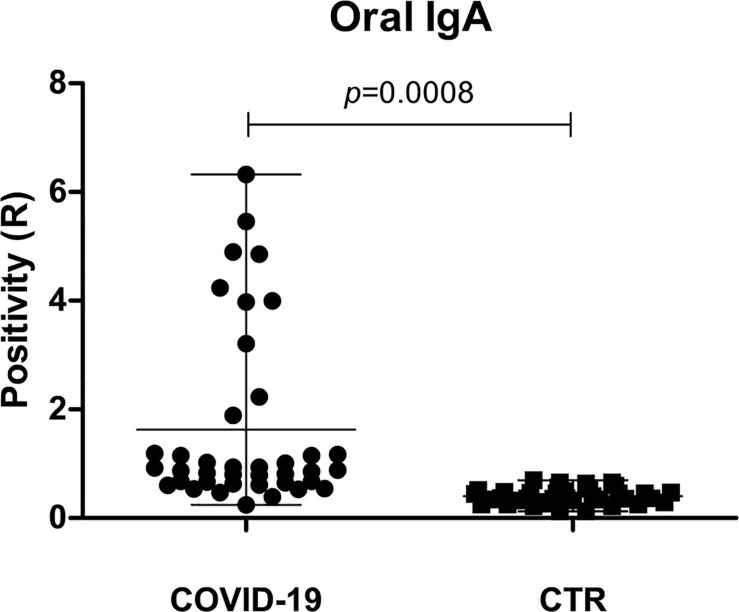
Mucosal sIgA response in the oral cavity of COVID-19 and control (CTR) subjects. The positivity is expressed as the ratio (R) between the value detected in the sample and the threshold control value, following manufacturer’s instructions; mean value with range is also shown.

To assess the development of a mucosal immune response against SARS-CoV-2 in the oral cavity, oral secretory IgA was searched and quantified by specific ELISA in the oral rinse samples of COVID-19 patients and controls. A mucosal IgA response was detected in 25/39 (64.1%) COVID-19 patients and no controls (*p* = 0.0008). Interestingly, the extent of mucosal response was different among the symptom subgroups of patients (Figure 7). In fact, 10/39 patients (25.6%) exhibited a very high concentration of sIgA (R > 2.0), whereas 15/39 patients (38.5%) had intermediate values (0.8 < R < 2.0), and 14/39 (35.9%) showed the presence of a barely detectable (R∼0.8 threshold value) or no sIgA response. Of note, 6/10 COVID-19 patients displaying high oral sIgA titer were asymptomatic/paucisymptomatic, evidencing a trend toward an inverse correlation between the salivary sIgA concentration and symptom severity (Spearman *r*−0.355; 95% CI −0.600 to 0.047; *p* = 0.02).

### Oral Cytokines in COVID-19 Patients

**FIGURE 8:**
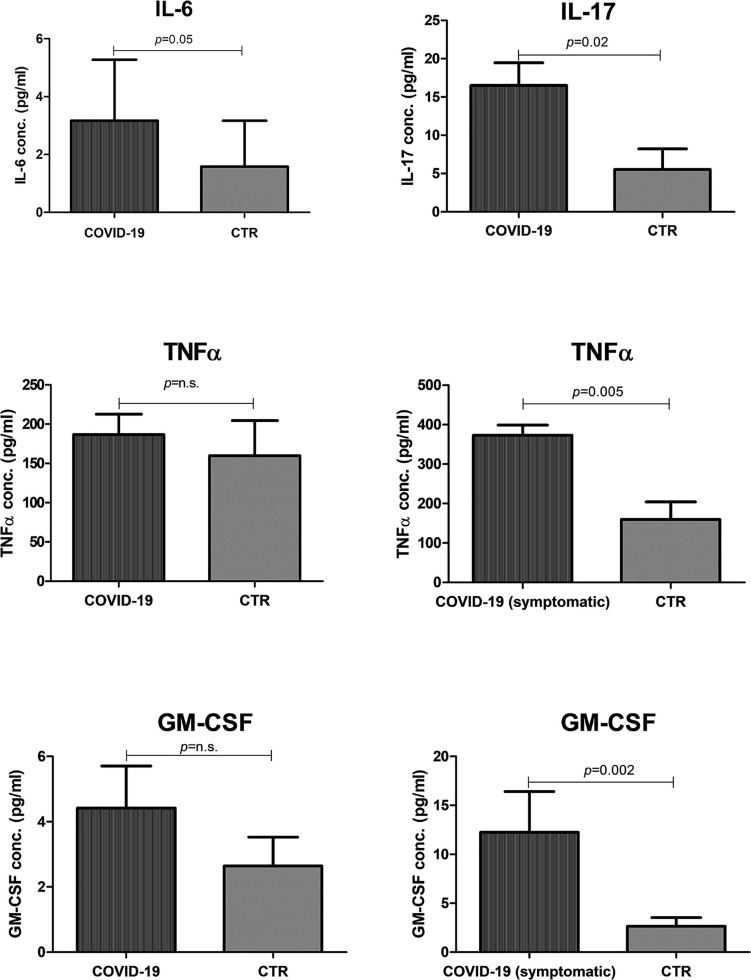
Presence of pro-inflammatory cytokines/chemokines in the oral cavity of COVID-19 patients and controls (CTR). The results are expressed as the mean values ± SEM of the concentration (pg/ml) for each indicated cytokine.

Since the so-called “cytokine storm” is a hallmark of severe COVID-19 disease, we investigated the release of pro-inflammatory cytokines in the oral cavity. Namely, the four main cytokines/chemokines detected in the blood of COVID-19 patients were analyzed: IL-6, IL-17, TNFα, and GM-CSF. The results showed that both IL-6 (*p* = 0.005) and IL-17 (*p* = 0.02) were significantly higher in COVID-19 oral samples than in controls (Figure 8). TNFα and GM-CSF were also more concentrated in COVID-19 patients compared to controls, but the differences were not statistically significant. However, the differences became significant by comparing COVID-19 symptomatic subgroup with controls (TNFα *p* = 0.005; GM-CSF *p* = 0.002), highlighting that more inflammation was detectable in the subjects undergoing a more severe course of the disease.

Inflammation also correlated with the oral microbiome dysbiosis, being more pronounced in subjects with a more evident decrease of alpha-diversity and species richness (*p* < 0.01).

## Discussion

Recent reports have shed light on the role of the microbiome in several diseases, including those of viral origin, suggesting that the commensal microbiota may potentially favor or hamper viral infections. However, most studies consider the gut microbiome, neglecting the role of an oral one. In addition, most if not all studies discuss only bacterial microbiota, whereas fungi and viruses are also important components of the commensal microbiota. Concerning SARS-CoV-2 infection, COVID-19 patients have been reported to harbor oral pathogenic bacteria (such as cariogenic or periodontopathic pathogens) (Bao et al., 2020; Patel and Sampson, 2020; Xiang et al., 2020). Oral dysbiosis might favor the establishment of SARS-CoV-2 infection through different mechanisms, as known for other respiratory viruses, including alteration of the respiratory epithelium, promotion of adhesion of respiratory pathogens, and increase of local inflammation (Baghbani et al., 2020). Despite such suggestions, the profile of the HOM is currently still not clarified, especially in the non-bacterial components, rendering it difficult to understand whether the HOM dysbiosis may be considered a risk factor for COVID-19 development (Patel and Sampson, 2020). Two recent preprints reported on the bacterial profile of HOM in COVID-19 patients, suggesting relationships between some bacteria and SARS-CoV-2 infection (Iebba et al., 2020; Ward et al., 2021). However, to date, no studies have completely addressed HOM profiling, including fungal and viral components.

Thus, our study aimed to characterize by metagenomics (WGS deep sequencing) the oral microbiome of COVID-19, to get a comprehensive view of its bacterial, fungal, and viral components.

The results showed very significant differences in the HOM composition between COVID-19 and control subjects, highlighting a decrease in the alpha-diversity and bacterial species richness in COVID-19 patients compared to controls, and a significant correlation between such decrease and symptom severity (*p* = 0.006). These data are in line with previous observations highlighting a decrease in the alpha variety and species richness upon HCV, HIV, and influenza infection (Sun et al., 2016; Inoue et al., 2018), with a parallel increase of pro-inflammatory cytokines like IL-6, TNFα, and IL-1β (Yildiz et al., 2018; Ramos-Sevillano et al., 2019).

Our results also showed an increase in the relative abundance of genera associated with poor oral hygiene and periodontitis in COVID-19 patients (*Prevotella*, *Lactobacillus*, *Capnocytophaga, Porphyromonas, Abiotrophia, Aggregatibacter*, and *Atopobium*), suggesting an association between those bacteria and SARS-CoV-2 infection, similar to that reported for other respiratory viruses (Andrews et al., 2012; Wang et al., 2016). The exclusive presence of *Enterococcus* and *Enterobacter* genera in COVID-19 patients suggests that they might be a microbial marker of susceptibility for SARS-CoV-2 infection. Even more interesting, fungi were instead more abundant in COVID-19 patients than in controls, with some genera (*Aspergillus*, *Nakaseomyces*, and *Malassezia*) only detectable in COVID-19 subjects, besides the more common *Candida* and *Saccharomyces* genera. In this regard, significant differences in fungal community with a higher richness of fungal species were detected in HIV-infected compared to uninfected individuals (Mukherjee et al., 2014) and in HBV/HCV symptomatic patients, where the diversity of intestinal fungi was positively associated with disease progression (Chen et al., 2011). Oral mycetes may be increased in the mouth because of bacterial alterations, ultimately favoring SARS-CoV-2 infection, due to the increased inflammation originated by fungi enzymatic and catabolic/toxic activity in the mouth (Chen X. et al., 2020). Beyond the potential mechanisms underlying the cooperation between SARS-CoV-2 and fungi, the results suggest that it could be important to consider this component of HOM in the management of virus infection.

Another non-bacterial HOM component that was augmented in COVID-19 patients was the viral one (from 0.07 to 1.12% of the total microbiome). HSV-1 and EBV herpesviruses were most present, and EBV coinfection was evidenced in about 30% of COVID-19 patients compared to only 5% of controls. In this regard, the HOM dysbiosis may have facilitated the activation/reactivation of oral viruses, and in turn, the high presence of herpesviruses infection/reactivation may further impair proper immune control (Jasinski-Bergner et al., 2020), thus potentially contributing to worse efficiency of the immune response against SARS-CoV-2. Consistent with this, EBV infection was detected in COVID-19 patients, associated with increased risk of severe COVID-19 symptoms and fatal outcome (Roncati et al., 2020; Chen et al., 2021), and correlated increased levels of IL-6 (Lehner et al., 2020). Similarly, alpha-herpesvirus (HSV-1, VZV) reactivation was observed, impacting the prognosis of COVID-19 patients (Le Balc’h et al., 2020; Hernandez et al., 2021). Thus, the presence of Herpesviridae infections in the oral cavity and their direct consequences deserve further investigation.

In parallel with the HOM profile, our work also characterized the local inflammatory and immune response as critical parameters to understand the evolution of the SARS-CoV-2 infection at the primary site of entry.

A hallmark of disease severity in COVID-19 is the uncontrolled inflammatory response, with the detection of IL-6, IL-17, TNFα, and GM-CSF at the serum/blood level (Chen G. et al., 2020; Mehta et al., 2020; Parra-Medina et al., 2020), the so-called “cytokine storm.” Here we showed a significant increase of those cytokines in the oral cavity of COVID-19 patients, indicating the development of inflammation right at the entry site of the virus. It is noteworthy that the level of oral inflammation paralleled the symptom severity, pointing to the importance of oral conditions for the subsequent systemization of virus infection and inflammation cascade.

A still unanswered point in COVID-19 progression regards the development and role of the local immune response against SARS-CoV-2. Mucosal sIgA has long been known to be crucial in controlling viruses that enter the body via mucosal surfaces (Yan et al., 2002); sIgA were indeed found in the ocular fluid of at least 40% of COVID-19 patients (Caselli et al., 2020b), and microbiome composition is reportedly known to interact with and influence IgA response, in different anatomical niches including the nares (Salk et al., 2016; Grosserichter-Wagener et al., 2019; Pabst and Slack, 2020). Here, we demonstrate anti-SARS-CoV-2 sIgA in the oral cavity and that they are significantly more abundant in asymptomatic/paucisymptomatic COVID-19 patients (*p* = 0.02), suggesting that sIgA may be important in controlling virus penetration in the body.

The main limitation of our study is the number of enrolled subjects, who represented all the eligible subjects hosted at the enrolled center. The enrollment of a higher number of subjects, ideally in a multi-center study, may confirm the generalizability of the study results. A higher number of subjects would also enable us to stratify patients for age, thus providing a direct comparison of more homogeneous microbial populations, as the microbiome composition is dependent on the subject’s age (Bourgeois et al., 2017; Caselli et al., 2020a). The relatively low number of recruited patients in our study also did not enable us to evidence a high statistically significant correlation between sIgA production and protection from severe COVID-19. Thus, studying a higher number of patients may be of importance to ascertain this point, especially in developing effective prevention strategies and vaccines.

Overall, the data presented here suggest a correlation between HOM dysbiosis and individual susceptibility to SARS-CoV-2 severe infection, indicating an interplay between HOM profile (including mycobiome and virome), inflammation, and mucosal IgA response. If HOM alteration is the cause or effect of severe COVID-19, it is not currently possible to distinguish, because the presence of SARS-CoV-2 in the oral cavity may impact microbiome dysbiosis (Xiang et al., 2020; de Oliveira et al., 2021). On the other hand, connections between oral dysbiosis and post-viral complications have been reported, suggesting that improving oral health may reduce the risk of complications from COVID-19 (Sampson et al., 2020), thus supporting the hypothesis of a role of dysbiosis in the virus-induced disease. Toward this direction, recent studies reported that SARS-CoV-2 load can be reduced by the use of chlorhexidine mouthwashes (Yoon et al., 2020), supporting the use of antiseptics against coronavirus infection (Koch-Heier et al., 2021; Mateos Moreno et al., 2021), and clinical studies are developing accordingly (Carrouel et al., 2020) and hopefully will help to clarify this aspect.

These findings may be important in defining markers useful to predict the development of symptomatic COVID-19, and open new therapeutic opportunities addressed to balance HOM and inflammation to prevent the development of severe symptoms. In this direction, IL-6 inhibitors have been reported to reduce the odds of COVID-19 mortality (Sinha et al., 2021), and specific probiotic administration has been proposed to balance microbiome dysbiosis and prevent the development of virus-induced respiratory diseases (Wang et al., 2016) and may represent a possible intervention in COVID-19 patients.

## Data Availability Statement

The datasets presented in this study can be found in online repositories. The names of the repository/repositories and accession number(s) can be found below: https://www.ebi.ac.uk/ena, PRJEB42999.

## Ethics Statement

The studies involving human participants were reviewed and approved by the Ethics Committee Area Vasta Emilia Centro della Regione Emilia-Romagna (CE-AVEC): approval document no. 408/2020/Oss/UniFe, approved on April 21, 2020. The patients/participants provided their written informed consent to participate in this study.

## Author Contributions

IS and MD’A analyzed the samples. CF and MF contributed to the design of the study, protocol for oral sampling, and interpretation of data. AP provided COVID-19 clinical samples and patient data. RM and GZ provided COVID-19 clinical samples and interpretation of clinical data. ML and CC provided control clinical samples. CC contributed to writing the manuscript. EC designed the study, elaborated the results, and wrote the manuscript. All authors read and approved the final manuscript.

## Conflict of Interest

The authors declare that the research was conducted in the absence of any commercial or financial relationships that could be construed as a potential conflict of interest.
